# Impact of CD34^+^ cell dose on outcomes of haploidentical peripheral blood stem cell transplantation in acute leukemia

**DOI:** 10.1007/s44313-025-00091-5

**Published:** 2025-08-07

**Authors:** Haerim Chung, Hye Won Kook, Hyunsoo Cho, Ji Eun Jang, June-Won Cheong

**Affiliations:** 1 Division of Hematology, Department of Internal Medicine, Severance Hospital, Yonsei University College of Medicine, Yonsei-ro 50-1, Seodaemun-gu, Seoul, 03722 Republic of Korea; 2 Blood Cancer Research Institute, Yonsei University College of Medicine, Seoul, 03722 Republic of Korea

**Keywords:** Haploidentical stem cell transplantation, CD34^+^ stem cell dose, Graft host versus disease, Non-relapse mortality

## Abstract

**Purpose:**

Allogeneic hematopoietic stem cell transplantation remains a curative option for acute leukemia. While an adequate CD34^+^ cell dose is essential for engraftment, the optimal upper threshold in haploidentical peripheral blood stem cell transplantation (haplo-PBSCT) remains unclear.

**Methods:**

We retrospectively analyzed 81 patients with acute leukemia who underwent haplo-PBSCT with reduced-intensity conditioning between 2010 and 2020. Patients were stratified by CD34^+^ cell dose (< 8 × 10^6^/kg vs. ≥ 8 × 10^6^/kg). Clinical outcomes, including overall survival (OS), non-relapse mortality (NRM), graft failure, and graft-versus-host disease (GVHD) incidence, were compared.

**Results:**

A higher CD34^+^ cell dose was associated with inferior OS (*P* = 0.022) and increased NRM (*P* = 0.002), despite similar rates of graft failure and acute GVHD. Chronic GVHD was more frequent in the higher dose group, though the difference was not statistically significant. Multivariate Cox analysis confirmed a high CD34^+^ cell dose as an independent predictor of poor OS (HR 2.054, *P* = 0.031).

**Conclusion:**

These findings suggest that excessively high doses may adversely affect survival by increasing transplant-related toxicity. Graft cell dose should be carefully balanced to optimize outcomes in haplo-PBSCT.

## Introduction

Allogeneic hematopoietic stem cell transplantation (allo-HSCT) remains a cornerstone in the treatment of hematologic malignancies and, for many patients, represents the only curative option. By replacing the patient’s diseased or damaged bone marrow with healthy hematopoietic stem cells from a compatible donor, allo-HSCT offers the potential for long-term remission and cure. It is particularly effective in conditions such as acute leukemias, myelodysplastic syndromes, and certain lymphomas, where both the graft-versus-tumor effect and complete hematopoietic reconstitution play vital roles in disease eradication [1, 2]. Despite substantial risks—including graft-versus-host disease (GVHD), infections, and regimen-related toxicity—allo-HSCT continues to offer potentially life-saving outcomes [3].

Among the many variables that influence transplant outcomes, the dose of infused stem cells is a critical determinant. The stem cell dose is typically quantified by the number of CD34^+^ hematopoietic progenitor cells per kilogram of the recipient’s body weight. CD34^+^ cells are widely accepted as a surrogate marker for stem cell content, and their quantity significantly influences engraftment, immune recovery, and post-transplant complications [4]. An adequate CD34^+^ cell dose is essential to ensure successful and timely engraftment. Insufficient doses have consistently been associated with delayed engraftment, prolonged cytopenia, and an increased risk of graft failure  [5]. There is a relatively well-established consensus regarding the minimum required CD34^+^ cell dose to ensure reliable engraftment. Most clinical guidelines and studies recommend a threshold of at least 2 × 10^6^ CD34^+^ cells/kg, below which the risk of delayed neutrophil and platelet recovery significantly increases [6]. However, while higher stem cell doses may accelerate engraftment, they have also been linked to an increased risk of GVHD, particularly in peripheral blood stem cell transplants [7].

In the setting of allo-HSCT using haploidentical donors, the optimal stem cell dose remains a subject of ongoing debate. Some studies suggest that higher stem cell doses may be beneficial by promoting engraftment and immune reconstitution [8], whereas others report an association between high stem cell doses and adverse post-transplant outcomes [9]. Owing to variability in transplant platforms, conditioning regimens, and graft manipulation techniques, there is currently no consensus on the ideal CD34^+^ cell dose in haploidentical peripheral blood stem cell transplantation (haplo-PBSCT). In this study, we aimed to evaluate the clinical significance of CD34^+^ cell dose in haplo-PBSCT, with particular attention to the prognostic implications of higher-dose infusion.

## Patients and methods

### Patients

All 95 patients with acute leukemia who underwent their first haplo-PBSCT at Severance Hospital, Yonsei University College of Medicine, between 2010 and 2020 were initially enrolled in this study. Fourteen patients who received myeloablative conditioning chemotherapy were excluded. A total of 81 patients who received reduced-intensity conditioning (RIC) regimens—consisting of either fludarabine with busulfan or fludarabine with cyclophosphamide—were retrospectively analyzed. All patients received a calcineurin inhibitor as part of GVHD prophylaxis. Among them, 67 patients were administered anti-thymocyte globulin (ATG) at a total dose of 7.5–10 mg/kg, while the remaining 14 patients received post-transplant cyclophosphamide (PTCy) at 50 mg/kg on days + 3 and + 4.

This study was approved by the Institutional Review Board of Severance Hospital (IRB No. 4–2010–0669) and was conducted in accordance with the Declaration of Helsinki. The cohort includes both prospective and retrospective data and is registered at ClinicalTrials.gov (NCT02344953). Written informed consent was obtained from all participants, and all patient samples were coded and anonymized.

### Transplantation

All patients underwent high-resolution HLA typing at the allele level for the HLA-A, -B, -C, and -DR loci. Patients without matched sibling donors or matched unrelated donors from domestic stem cell donor registries—the Korea Marrow Donor Program and the Catholic Hemopoietic Stem Cell Bank—underwent haploidentical transplantation. Among haploidentical donors, final donor selection was determined through family meetings, prioritizing factors such as donor age, underlying medical conditions, and ABO incompatibility.

All recipients received T cell–replete peripheral blood stem cell (PBSC) grafts. Stem cells were mobilized using subcutaneous granulocyte colony-stimulating factor (G-CSF) administered daily for 4 to 5 days. In all cases, the CD34^+^ cell dose was measured only after graft collection. The entire PBSC graft collected during apheresis was infused into the recipient, regardless of the CD34^+^ cell count. In accordance with institutional policy, a minimum CD34^+^ cell dose of 2 × 10^6^ cells/kg is targeted for allo-HSCT, and all patients in this study received a dose meeting or exceeding this threshold.

The median CD34^+^ cell dose was 7.7 × 10^6^ cells/kg (range, 2.95–20 × 10^6^ cells/kg). To determine the optimal cutoff value for predicting overall survival (OS), we employed a binary partitioning method based on receiver operating characteristic curve analysis. This method identifies the cutoff point that maximizes sensitivity and specificity for OS discrimination. Using this approach, the cutoff value was determined to be 7.94 × 10^6^ cells/kg. For clinical applicability and standardization, the cutoff was rounded to 8 × 10^6^ cells/kg. The cohort was subsequently divided into two groups: 46 patients (56.8%) who received grafts with a CD34^+^ cell dose < 8 × 10^6^/kg were categorized as the adequate dose group, and 35 patients (43.2%) who received grafts with a CD34^+^ cell dose ≥ 8 × 10^6^/kg were categorized as the higher dose group.

### Statistical analysis

Group comparisons were performed using the χ^2^ test for categorical variables and the t test for continuous variables. Non-relapse mortality (NRM) was defined as death without evidence of disease relapse or progression, calculated from the date of transplantation. Univariate and multivariate analyses for OS and NRM were conducted using the Cox proportional hazards regression model. Two-sided *P* values < 0.05 were considered statistically significant. All statistical analyses were performed using SPSS version 28 (IBM Corp., Armonk, NY, USA).

## Results

### Patients and donor characteristics

A total of 81 patients with acute leukemia received haplo-PBSCT during the study period. The median follow-up duration for all patients was 19 months (range, 1.1–31.4 months).

Baseline characteristics of the entire cohort, stratified by CD34^+^ cell dose, are summarized in Table 1. No significant differences were observed between the two groups in terms of major clinical parameters, including sex, underlying disease, disease status at transplantation, and Hematopoietic Cell Transplantation–Comorbidity Index (HCT-CI) scores. However, statistically significant differences were found in both recipient and donor age. Recipients in the higher CD34^+^ cell dose group were significantly older than those in the adequate dose group (*P* = 0.005). In contrast, donors in the higher dose group were significantly younger than those in the adequate dose group (*P* = 0.002). Other variables, such as the proportion of female-to-male transplants and use of ATG, were comparable between the two groups.

**Table 1:** Patient and transplant characteristics according to CD34^+^ cell dose

	All (*N* = 81)	≥ 8.0 × 10^6^/kg (*n* = 35)	< 8.0 × 10^6^/kg (*n* = 46)	*P* value
Recipient age, median (range)	45 (20–69)	53 (21–66)	40 (20–69)	0.005
Sex				NS
Male, n (%)	49 (60.5%)	19 (54.3%)	30 (65.2%)	
Disease				NS
AML n (%)	43 (53.1%)	20 (57.1%)	23 (50.0%)	
ALL, n (%)	27 (33.3%)	8 (22.9%)	19 (41.3%)	
MDS/MPN, n (%)	11 (13.6%)	7 (20.0%)	4 (8.7%)	
Disease status				NS
1 st CR, n (%)	66 (81.5%)	29 (82.9%)	37 (80.4%)	
Advanced, n (%)	15 (18.5%)	6 (17.1%)	9 (19.6%)	
HCT-CI score				NS
> 4, n (%)	16 (19.8%)	9 (25.7%)	7 (15.2%)	
Donor age, median (range)	37 (15–69)	33 (15–59)	46(15–69)	0.002
Sex mismatch				NS
Female to male, n (%)	22 (27.2%)	7 (20.0%)	15 (32.6%)	
GVHD prophylaxis				NS
ATG, n (%)	67 (82.7%)	29 (82.9%)	38 (82.6%)	
PTCy, n (%)	14 (17.3%)	6 (17.1%)	8 (17.4%)	

### Impact of CD34^+^ cell dose on main transplant outcomes

The key transplantation outcomes are summarized in Table 2. The incidence of graft failure was similar between the two groups (8.6% vs. 6.5%). Overall, 38 patients (46.9%) experienced acute GVHD of any grade during follow-up. The incidence of acute GVHD was not significantly different between the groups (*P* = 0.478), suggesting no clear association between CD34^+^ cell dose and the development of clinically significant acute GVHD. Among 69 patients (85.2%) who survived relapse-free beyond day 100 and were evaluable for chronic GVHD, the incidence of moderate-to-severe chronic GVHD was 31.9%. Notably, chronic GVHD was more frequently observed in the higher CD34^+^ cell dose group (46.2%) compared to the adequate CD34^+^ cell dose group (23.3%) (*P* = 0.048), suggesting a potential association between increased stem cell dose and a higher risk of chronic GVHD.

**Table 2:** Main post-transplant outcomes according to CD34^+^ cell dose

	All (*N* = 81)	≥ 8.0 × 10^6^/kg (*n* = 35)	< 8.0 × 10^6^/kg (*n* = 46)	*P* value
Graft failure, n (%)	6 (7.4%)	3 (8.6%)	3 (6.5%)	0.727
Acute GVHD, n (%)	38 (46.9%)	18 (51.4%)	20 (43.5%)	0.478
Chronic GVHD, n (%)	22/69 (31.9%)	12/26 (46.2%)	10/43 (23.3%)	0.048
Relapse, n (%)	22 (27.2%)	5 (14.3%)	17 (37.0%)	0.023
Non-relapse mortality, n (%)	23 (28.4%)	16 (45.7%)	7 (15.2%)	0.003
Infection	14 (17.3%)	10 (28.6%)	4 (8.7%)	—
GVHD, n (%)	5 (6.2%)	4 (11.4%)	1 (2.2%)	—
Overall mortality, n (%)	38 (46.9%)	21 (60.0%)	17 (37.0%)	0.040

With a median follow-up of 19 months, 38 patients (46.9%) had died, and 22 patients (27.2%) experienced disease relapse. The most common causes of NRM were infection (*n* = 14) and GVHD (*n* = 5). The overall mortality rate was significantly higher in the higher CD34^+^ cell dose group (60.0%) compared to the adequate dose group (37.0%) (*P* = 0.040), and NRM was also higher in the higher dose group (45.7%) than in the adequate dose group (15.2%) (*P* = 0.003). In contrast, relapses occurred more frequently in the adequate dose group (21.7%) than in the higher dose group (14.3%) (*P* = 0.023).

**Fig. 1:**
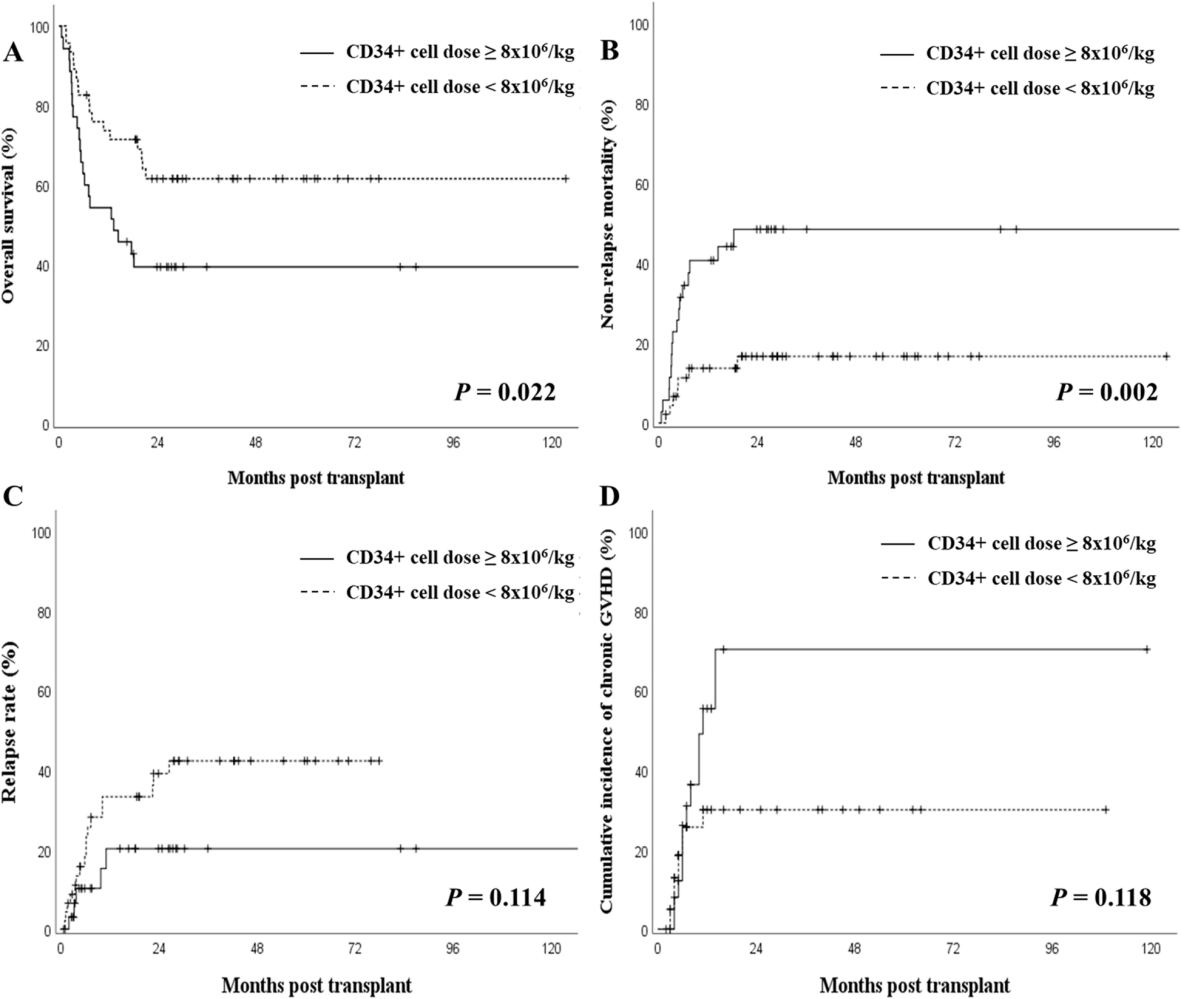
Overall survival (**A**), non-relapse mortality (**B**), relapse rate (**C**), and cumulative incidence of chronic GVHD (**D**) according to CD34^+^ cell dose

These findings were further supported by Kaplan–Meier and cumulative incidence analyses (Fig. 1). Patients who received a higher CD34^+^ cell dose demonstrated significantly inferior OS compared to those who received an adequate dose (*P* = 0.022; Fig. 1A). Moreover, NRM was significantly higher in the higher dose group (*P* = 0.002; Fig. 1B). Although not statistically significant, there was a trend toward an increased relapse rate in the adequate dose group (*P* = 0.114; Fig. 1C). The cumulative incidence of chronic GVHD appeared higher in patients receiving a higher CD34^+^ cell dose; however, this difference did not reach statistical significance (*P* = 0.118; Fig. 1D).

### Factors associated with OS and NRM

To identify risk factors associated with OS and NRM, analyses were performed using the Cox proportional hazards regression model (Table 3). In the univariate analysis, advanced disease status (hazard ratio [HR] 2.094, 95% confidence interval [CI] 1.011–4.338, *P* = 0.047), an HCT-CI score greater than 4 (HR 2.193, 95% CI 1.103–4.363, *P* = 0.025), and a higher CD34^+^ cell dose (HR 2.058, 95% CI 1.083–3.909, *P* = 0.028) were significantly associated with inferior OS. Recipient and donor age were not identified as significant prognostic variables. Multivariate analysis confirmed a higher CD34^+^ cell dose as an independent risk factor for worse OS (HR 2.054, 95% CI 1.067–3.954, *P* = 0.031). Similarly, in the univariate analysis for NRM, a higher CD34^+^ cell dose was identified as a significant independent predictor (HR 3.631, 95% CI 1.489–8.850, *P* = 0.005). However, neither advanced disease status (HR 1.863, 95% CI 0.731–4.747, *P* = 0.193) nor an HCT-CI score greater than 4 (HR 1.949, 95% CI 0.799–4.755, *P* = 0.143) were significantly associated with increased NRM risk.

**Table 3:** Univariate and multivariate analyses for overall survival and non-relapse mortality

Variable	Overall survival HR (95% CI)	*P*-value	Nor-relapse mortality HR (95% CI)	*P* value
Univariate anaylsis				
Recipient age	1.018 (0.994–1.043)	0.139	1.028 (0.996–1.061)	0.089
Advanced disease status	2.094 (1.011–4.338)	0.047	1.863 (0.731–4.747)	0.193
HCT-CI > 4	2.193 (1.103–4.363)	0.025	1.949 (0.799–4.755)	0.143
Donor age	0.991 (0.970–1.013)	0.438	1.005 (0.978–1.032)	0.736
Female donor to male recipient	1.325 (0.668–2.627)	0.421	1.227 (0.504–2.983)	0.652
High CD34^+^ cell dose (≥ 8 × 10^6^/kg)	2.058 (1.083–3.909)	0.028	3.631 (1.489–8.850)	0.005
Multivariate analysis				
Advanced disease status	2.147 (1.020–4.523)	0.044	—	—
High CD34^+^ cell dose (≥ 8 × 10^6^/kg)	2.054 (1.067–3.954)	0.031	—	

## Discussion

Our analysis revealed a paradoxical impact of CD34^+^ stem cell dose on haplo-PBSCT outcomes in acute leukemia. Notably, patients who received an excessively high CD34^+^ cell dose (≥ 8 × 10^6^/kg) experienced significantly inferior OS, primarily driven by increased NRM, compared to those who received adequate doses**.** The relationship between graft CD34^+^ cell dose and transplant outcomes has been debated in the literature. Several studies, particularly in the context of HLA-matched transplantation, have reported that higher CD34^+^ cell doses may improve outcomes by enhancing engraftment kinetics and reducing both NRM and relapse rates [10]. Conversely, other studies have raised concerns that excessively high CD34^+^ cell doses may increase the risk of GVHD, particularly chronic GVHD, thereby offsetting those benefits. Mohty et al. (2003) reported that in HLA-identical sibling transplants, CD34^+^ cell doses exceeding 8 × 10^6^/kg were associated with increased mortality attributable to chronic GVHD [7]. Similarly, Dhedin et al. (2012) observed that higher donor CD34^+^ counts after G-CSF mobilization correlated with a greater incidence of extensive chronic GVHD in reduced-intensity conditioning PBSC transplants [11]. These findings are consistent with our observation of a trend toward increased chronic GVHD and higher NRM in the higher CD34^+^ dose group, suggesting that the adverse effects of large graft doses may be applicable across diverse transplant platforms.

In the specific context of haplo-PBSCT, published data are somewhat inconsistent. Pedraza et al. (2022) [12] evaluated the impact of CD34^+^ cell dose in PTCy–based transplants across various donor types and found a significant association only in haplo-PBSCT. Patients who received lower CD34^+^ cell doses (≤ 5 × 10^6^/kg) experienced significantly worse survival compared to those who received > 5 × 10^6^/kg. In their study, which capped the maximum CD34^+^ cell dose at 8 × 10^6^/kg, higher doses were associated with improved outcomes, likely due to more rapid engraftment and enhanced graft-versus-leukemia (GVL) effects. Our findings differ, as we observed adverse outcomes at the upper extreme of CD34^+^ dosing (≥ 8 × 10^6^/kg). This discrepancy suggests the existence of an optimal CD34^+^ cell dose range, where moderate increases are beneficial, but doses beyond a certain threshold may lead to harm.

Recent large-scale data support the hypothesis that the relationship between CD34^+^ cell dose and transplant outcomes is non-linear. A Japanese retrospective study in the setting of haploidentical PBSCT with PTCy demonstrated that the lowest mortality risk occurred at a CD34^+^ cell dose of approximately 5 × 10^6^/kg, with both lower and higher doses associated with increased risks [13]. Notably, while this study did not report a significant association between extreme CD34^+^ cell doses and either NRM or GVHD incidence, it did identify higher relapse rates at both ends of the dosing spectrum. Our findings are partially consistent with these results, particularly in suggesting that intermediate CD34^+^ cell doses may be optimal. However, our study uniquely identified a significant increase in NRM among patients receiving high CD34^+^ cell doses (≥ 8 × 10^6^/kg). This discrepancy may be attributable to the higher proportion of patients in our cohort who received doses above 8 × 10^6^/kg, potentially uncovering an adverse NRM signal not detected in previous studies with narrower dosing ranges. This observation highlights the need to more precisely define an upper threshold beyond which additional CD34^+^ cells may be detrimental.

In line with institutional policy, a minimum CD34^+^ cell dose of 2 × 10^6^ cells/kg is targeted to ensure adequate engraftment in allo-HSCT. Importantly, our study did not include any patients who received doses below this threshold. Therefore, the present analysis focuses on outcomes associated with adequate to high CD34^+^ cell doses and does not address the impact of suboptimal graft doses.

The detrimental impact of an excessively high CD34^+^ cell dose may reflect the complex immunologic composition of the graft. Apheresis products with elevated CD34^+^ counts typically contain proportionally higher numbers of lymphocytes—CD3^+^ T cells, natural killer cells—and other immunologically active cells. In T cell–replete haplo-PBSCT, these alloreactive cells are generally controlled by PTCy or immunosuppressive therapy [14]. However, infusion of a very large graft may exceed the capacity of these protective mechanisms.

One hypothesis is that an increased T cell burden elevates the risk of residual alloreactive donor T cells, contributing to chronic GVHD. Although not statistically significant, chronic GVHD was more frequent in our high-dose group and is a recognized driver of late NRM [15]. This suggests that excess donor immune cells may promote persistent alloimmunity, tissue injury, and infection risk, thereby increasing NRM. Interestingly, we did not observe an increase in acute GVHD with higher CD34^+^ cell doses, consistent with previous reports. Some studies have even suggested that very high total nucleated cell doses may reduce acute GVHD, possibly due to the co-infusion of regulatory immune cells such as regulatory T cells or myeloid-derived suppressor cells that mitigate acute alloreactivity [16, 17]. Our findings may reflect this balance: while acute GVHD did not increase, a trend toward more chronic GVHD emerged.

This is biologically plausible, as chronic GVHD is driven by immune mechanisms distinct from those underlying acute GVHD. Whereas acute GVHD is primarily mediated by early activation of donor-derived cytotoxic T cells and pro-inflammatory cytokines, chronic GVHD involves a more complex interplay of adaptive and innate immune responses, leading to autoimmune-like manifestations and progressive tissue fibrosis [18]. Furthermore, larger graft sizes may enhance the recovery of these immune subsets, disrupt immune tolerance, and increase the presence of donor antigen-presenting cells, ultimately resulting in sustained alloimmune activation. These mechanisms contribute to tissue injury, fibrosis, and an elevated risk of infections, thereby increasing NRM rates.

Another important consideration is the GVL effect. In general, higher infused cell doses are thought to enhance GVL activity by increasing the frequency of donor immune effector cells. Indeed, several studies have reported an association between higher CD34^+^ cell doses and reduced relapse rates​ [19, 20]. In our study, although the adequate dose group showed a trend toward a higher relapse rate compared to the higher dose group, the inferior OS observed in the higher dose group suggests that any potential reduction in relapse may have been offset by an increase in NRM. These findings imply that once the CD34^+^ cell dose exceeds a certain threshold, the cumulative toxicity of the transplant may outweigh its therapeutic benefits, leading to diminished leukemia control and an increased risk of complications. Therefore, our results highlight the critical importance of achieving a balance in allo-HSCT by administering a sufficient graft to ensure disease control and engraftment, while avoiding excessive cell doses that may provoke GVHD or other immune-mediated toxicities.

In our study cohort, donors in the higher CD34^+^ cell dose group tended to be younger, consistent with previous findings that older donors generally yield lower CD34^+^ cell counts during collection [21]. However, neither patient nor donor age independently predicted GVHD or NRM in our analysis. Notably, despite younger donor age, the higher CD34^+^ cell dose group exhibited increased rates of chronic GVHD and NRM, which contrasts with several recent studies suggesting that younger donors are associated with a lower risk of these complications [22, 23]. Patient-related factors such as age and comorbidities also did not emerge as significant predictors in our multivariate model. While advanced age and higher HCT-CI scores are generally associated with increased NRM in allo-HSCT, this was not observed in our haploidentical cohort—possibly reflecting improvements in supportive care and transplant protocols. In particular, the use of RIC regimens may mitigate the adverse impact of older age and comorbidities, thereby allowing broader eligibility for transplantation.

Like all retrospective analyses, our study has several limitations that warrant consideration. First, the data were collected retrospectively at a single institution, which may introduce inherent biases in patient and donor selection, supportive care, and clinical management strategies. As a result, the findings may not be fully generalizable to other centers with differing transplant protocols and practices. Second, although all patients underwent RIC haplo-PBSCT, there were variations in GVHD prophylaxis regimens, which could have influenced outcomes such as GVHD incidence and NRM. While both ATG and PTCy are effective in preventing chronic GVHD, PTCy appears to be associated with more favorable outcomes in terms of survival and NRM, particularly in mismatched donor settings [24]. However, in our study, due to the limited sample size, we could not identify statistically significant differences in clinical outcomes according to the type of GVHD prophylaxis. Future studies involving a larger, more homogeneous cohort receiving a standardized GVHD prophylaxis regimen are warranted to better evaluate its impact.

In conclusion, our findings suggest that a higher CD34^+^ cell dose does not inherently confer improved outcomes in the setting of haplo-PBSCT. While an adequate CD34^+^ cell dose remains essential for ensuring engraftment and disease control, excessive doses greater than 8 × 10^6^ CD34^+^ cells/kg may be associated with diminishing clinical benefit and increased transplant-related toxicity, particularly through elevated risks of chronic GVHD and NRM. These findings highlight the necessity for prospective studies and further investigation to define the optimal CD34^+^ dosing threshold. In the context of precision transplant medicine, tailoring graft cell dose according to patient-specific factors, disease characteristics, and transplant platforms may represent a key strategy for maximizing therapeutic efficacy while minimizing treatment-related complications.

## Data Availability

The datasets generated and analyzed during the current study are available from the corresponding author on reasonable request.
